# Acute dermatitis in adult female patients receiving hypofractionated radiotherapy for breast cancer: experience from a low- and middle-income country

**DOI:** 10.3332/ecancer.2022.1412

**Published:** 2022-06-15

**Authors:** Yumna Ahmed, Agha Muhammad Hammad Khan, Fatima Shaukat, Rabia Tahseen, Maria Tariq, Bilal Mazhar, Sehrish Abrar, Nasir Ali

**Affiliations:** Department of Radiation Oncology, Aga Khan University Hospital, Karachi 74800, Pakistan

**Keywords:** hypofractionated, breast cancer, radiation, dermatitis, toxicity

## Abstract

Radiotherapy (RT) is an important component of treatment in the management of breast cancer patients. The radiation treatment paradigm has been shifted towards hypofractionated RT. This study aims to determine the severity of acute dermatitis in patients receiving hypofractionated RT for breast cancer at a tertiary care university hospital in Pakistan. Patients with biopsy-proven invasive breast carcinoma or DCIS who were referred for radical radiotherapy after discussion in the breast tumour board were retrospectively reviewed. Physical assessment of the patients for evaluation of the severity of radiation dermatitis will be carried out in the first week, last week and on the first follow-up after 1 month of completion of RT, according to the Radiation Therapy Oncology Group/European Organisation For Research And Treatment Of Cancer (RTOG/EORTC) criteria. We identified 92 female patients in 6 months at Aga Khan University Hospital, with a mean age of 53.1 years. Most of the treated patients had clinical stage 3 (64%) cancer, while others were stage 2 (42%), stage 1 (2%) and stage 0 (2%). The surgeries performed were mastectomy in 59 patients and breast-conserving surgery in 33 patients. Histology was Intra Ductal Carcinoma (IDC) (95%), DCIS (3%) and Invasive Lobular Carcinoma (ILC) (2%). Most of the patients received chemotherapy (96%). Radiotherapy dose was 4256 cGy in 16 fractions, followed by a boost of 10 Gy. The radiation techniques used were intensity-modulated radiotherapy (47.8%) and three-dimensional conformal radiotherapy (52.2%). Most of the patients experienced no toxicity (59%), while grade I toxicity was observed in 29% of the patients and grade II toxicity was observed in 11%. Only 1% of the patients experienced grade III skin toxicity. Hypofractionated radiation therapy is beneficial because of the shorter overall treatment time which reduces the socio-economic burden, not only for patients but also for radiotherapeutic institutions. However, extended follow-up is to be reported for long-term toxicity and other consequences.

## Introduction

Breast cancer is the leading cause of morbidity and mortality in women globally [1, 2]. Pakistan has the highest incidence of breast cancer in Asia, with 1 in every 9 women at risk of developing breast cancer [3, 4]. According to a report, breast cancer accounts for about 34.6% of all cancer cases in Karachi [5], and Pakistani women present at advanced stages with more aggressive diseases. The late presentation may partly be due to a lack of awareness and knowledge about early symptoms and screening for risk factors [5]. Adjuvant radiotherapy (RT) after surgical resection of tumour improves loco-regional control and survival [6, 7]; hence, it is currently the standard of care for all women with breast cancer after breast conservation surgery (BCS) [8]. In early breast cancer (EBC), the 5-year local relapse rate dropped from 4.1% to 1.3% if radiation was offered in their management plan [9]. RT is generally a part of their treatment plan for locally advanced breast cancer (LABC) patients after mastectomy, especially in all T3 and axillary node-positive diseases [10].

Historically, the conventional regimen for adjuvant breast RT was 50 Gray (Gy), at 2 Gy per fraction. Recently, there has been an increasing global interest in the ‘hypofractionated’ regimen which delivers more than 2 Gy/fraction of radiation while reducing the total cumulative dose and reducing the number of treatment days favouring the low- and middle-income country (LMIC) [11]. The hypofractionated regimen offers similar clinical outcomes but fewer hospital visits, providing a practical and cost-effective treatment approach [12]. The skin is susceptible to damage by ionising radiation since it is a highly proliferative and self-renewing organ [13], hence nearly all patients who receive RT for breast cancer experience some degree of radiation dermatitis varying from mild to brisk erythema with or without moist desquamation and, occasionally, ulceration of the skin [14, 15]. They may suffer from dryness, redness, itching, burning, pain and discomfort, which may affect daily life activities and in severe cases, cause interruptions in treatment [16]. In an analysis of the toxicity of hypofractionated RT in breast carcinoma, all patients experienced some degree of acute dermatitis but the most commonly observed severity was grade I experienced by 80% of the patients, grade II by 18% and less than 2% had grade III and IV dermatitis, making it an ideal fractionated schedule in terms of the toxicity profile [12].

This study aims to determine the severity of acute dermatitis in adult female patients receiving hypofractionated radiation therapy for breast cancer to fill the gap in local data especially since the hypofractionated regimen potentially offers comparable outcomes with fewer hospital visits and lower costs in resource-constrained regions.

## Methodology

This is a single institution, retrospective chart review to determine the safety of hypofractionated radiation therapy as part of breast cancer treatment. Institutional Ethical Review Committee’s approval was obtained. All patients with biopsy-proven invasive carcinoma or *in situ* lesion arising from breast cancer, who were referred by a breast surgeon or medical oncologist for radical radiotherapy with a hypofractionated schedule, were enrolled in the study. The inclusion criteria were a) patients with non-metastatic breast cancer after breast-conserving surgery or mastectomy; b) age between 20 and 80 years; and (c) carcinoma *in situ* or invasive breast cancer proven by histology of the surgical specimen. Patients who had received previous radiotherapy in the same region or had a history of systemic lupus erythematosus or scleroderma and those with bilateral breast cancer were excluded. A report of 6 months from 1 December 2018 to 30 May 2019 was documented.

Considering 80% grade 1 toxicity from the previous studies, we enrolled 92 patients after (obtaining informed consent) for a 95% confidence level and 8% bound on error of estimation using non-probability consecutive sampling.

### Data collection

The acute phase of radiation dermatitis was observed from medical records for first and last week of treatment and on first follow-up after 1-month completion of radiation therapy.

During each visit, examination of the severity of reactions was graded from 0 to IV, according to the RTOG/EORTC acute radiation morbidity scoring criteria, version 2.0 [17].

Data were collected by the researcher from hospital medical records and documented as required in the study proforma.

### Radiation planning and delivery

A hypofractionated course of radiation in our study was defined as a definitive dose to the whole breast of 2.6 Gy/fraction. Patients were simulated with a computed tomography scan in the supine position. Our clinical pathway permitted optional boost radiotherapy of 10 Gy in 4–5 fractions using electron beam for post-mastectomy chest wall scar boost or photon beam lumpectomy boost radiotherapy in patients who underwent BCS. Treatment plans were created using 2D, 3D or intensity-modulated radiotherapy (IMRT) techniques as per the physician’s choice and considering patients’ financial constraints to meet the institutional dosimetric criteria, limiting WB-CTV V105 to 95% (95% of the PTV receiving 95% of the prescribed dose), although D90 >90% was also deemed acceptable to meet homogeneity goals.

### Data analysis

SPSS (version 21.0) was used for statistical analysis. A descriptive analysis was carried out. Mean and standard deviation were calculated for quantitative *variables*, i.e., age and frequency of occurrence of dermatitis. Percentages were calculated for qualitative variables, i.e., stage, type of surgery, chemotherapy, radiation technique and severity of acute dermatitis. Stratification of outcome variables was performed for effect modifiers like age, disease pathology, stage of disease, type of surgery, chemotherapy and radiation technique. Post-stratification chi-square test was applied. A *p*-value less than or equal to 0.05 was considered statistically significant.

## Results

92 patients received hypofractionated radiotherapy at our institution during this duration. The median age of female patients was 53.1 years (range = 22–80 years). An equal number of patients were treated for right and left breast cancers. Most patients had IDC 88 (95.7%) as the primary pathology. Most of the patients had stage III cancer (55.4, 51%), followed by stage II (37, 40.2%). One-third of the patients underwent BCS and received hypofractionated radiotherapy while two-thirds of the patients received radiation to the chest wall after mastectomy. Total radiation dose of 42.56 Gy at 2.66 Gy per day was delivered in all cases. A radiation boost of 10 Gy in 2 Gy daily fractions was delivered to 83 (90.2%) patients who were younger than 50 years of age, IDC grade 3 or positive margin disease. IMRT was the most used technique comprising 46 (50%) patients, followed by the three-dimensional conformal radiotherapy (3D CRT) technique in 44 (47.8%) patients. Two-dimensional (2D) was used in two (2.2%) patients. Most of our patients (89, 96%) already received chemotherapy before starting radiotherapy during the course of their treatment either in the neoadjuvant or adjuvant setting after definitive surgery. The patient and treatment characteristics are listed in Table 1.

### Early (1st week) treatment

**Figure 1.**
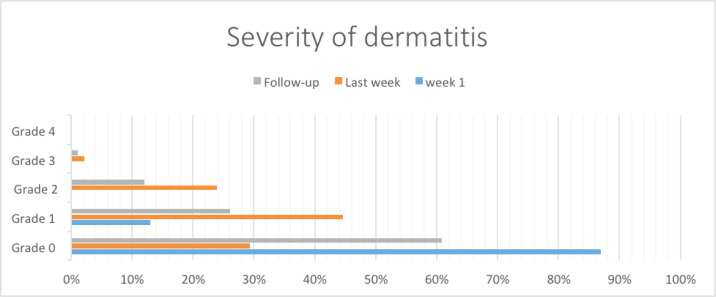
Bar chart illustrating the severity of dermatitis over time.

Most (80, 87%) patients had no dermatitis. Grade I dermatitis was present in 12 (13%) patients. None of the patients had grade II, III or IV dermatitis within the first week of radiotherapy (Figure 1).

### Late (last week) treatment

Grade 1 dermatitis was mostly reported during the last week of radiation therapy constituting 44% (*n* = 41) of the patients, while 24% (*n* = 22) had grade II dermatitis and approximately one-third of the patients experienced no toxicity (32%, *n* = 27).

### Post-treatment (1-month follow-up)

60% (*n* = 56) had no dermatitis at the post-treatment 1-month follow-up, while 26% (*n* = 24) had grade 1 dermatitis, 11% (*n* = 10) had grade 2 and 2.2% (*n* = 2) had grade 3 dermatitis.

### Stratification of severity of dermatitis with different variables

There was no significant difference in the severity of dermatitis during the first and last week of radiation and on follow-up after radiation relative to age and radiation boost. Grade 2 toxicity was significantly associated with the 3D CRT technique plan during the last week of radiotherapy (*p* = 0.05). However, no other association between severity of dermatitis and technique was found during the first week and on follow-up.

Grade 1 toxicity during follow-up after RT was significantly associated with mastectomy (*p* = 0.01). In a chest wall patient (undergoing mastectomy), the most common site of local recurrence is skin; so in these patients, a bolus is placed to increase the skin dose resulting in grade 2 skin dermatitis. Only patients with cT4 disease were found to be associated with grade 3 toxicity during follow-up (*p* = 0.03).

## Discussion

Breast cancer has the highest incidence among women worldwide. Approximately 90,000 new cases of breast cancer are diagnosed in Pakistan each year [18]. RT plays a core role in management, not only by reducing local recurrence but also by improving overall survival in both EBC and LABC patients [19]. In LMICs like Pakistan with limited public health resources and a significant lack of awareness about screening methods, which results in advanced stage presentation, RT treatment becomes a necessity for many with a long waiting list. A shift in adaptation to a hypofractionated schedule from the conventional was confirmed in a meta-analysis quoting 17 studies and concluded that there was no significant difference in local recurrence and improved toxicity and appearance outcomes noted in the hypofractionated with long-term follow-up [19, 20].

The first published phase II clinical trial of hypofractionated radiotherapy was published in 1987 with comparable loco-regional control and an acceptable cosmetic outcome [21].

A similar outcome was reported for 150 patients who underwent hypofractionated radiotherapy by Ortholan *et al* [22, 23]. Whelan et al also demonstrated the same finding in a better cohort of 1200 patients who received 50 Gy in 25 fractions or 42.56 Gy in 16 fractions with a 10-year follow-up. Grade 3 or higher acute dermatitis was only present in 3% of each group [24, 25]. A 12-year follow-up of a randomised

Canadian trial of 1234 women with early breast cancer reported the same outcome comparable between two groups of patients [26]. The Chinese randomised trial used hypofractionated RT in EBC patients who underwent breast-conserving surgery and showed no different toxicity than conventional arm [27]. All the studies mentioned above showed good tolerability of hypofractionated radiotherapy in breast cancer but cosmetic results have been somewhat mixed and likely depend on the specific schedule used [28]. Our study evaluated toxicity with a single regimen of hypofractionated radiotherapy and most of the patients had locally advanced diseases requiring chemotherapy. We offered a hypofractionated schedule to all our patients who had undergone BCS or modified radical mastectomy with the intention of reducing treatment time and comparing acute toxicity without compromising the oncologic result. The results from our study are consistent with international studies showing reduced skin toxicity with the hypofractionated RT schedule [29]. A total of 92 patients enrolled in our study were administered 2.66 Gy per fraction in an adjuvant setting after surgery and most of them did not develop toxicity in the first week. There is also insufficient data to evaluate the tolerability of shorter fractionation when used with different radiotherapy techniques, chemotherapy and type of surgical procedures [29]. There is a significant association between grade II and grade III dermatitis with 3D CRT and mastectomy, respectively. In addition to reduced toxicity, the hypofractionated schedule proved to be economically feasible because of the shorter overall treatment time, not only for patients but also beneficial for the limited number of hospitals providing radiation therapy to cancer patients in the region. Thus, implementation of shortened RT regimens would help decrease the burden on such institutions and help maintain mechanical and human resources required to meet the increasing demand for radiotherapy. Recently, further reduction of treatment time with a 1-week schedule of radiotherapy after primary surgery for EBC reported grade II as the most commonly observed acute toxicity (47%) as in our study and is proven safe in terms of normal tissue effects up to 5 years [30, 31]. The prevalence of acute dermatitis in our study suggested that severity is more intense during the last week of radiation (70%) and settled in one-third of the patients on follow-up after 4 weeks of radiation (28%). The grade III toxicity (2%) was only observed in patients who underwent mastectomy and had T4 disease so these patients should be kept under invigilation for clinical evaluation. However, since long-term follow-up was not done, only acute toxicity was assessed during and after completion of treatment. The impact of hypofractionated regimen on loco-regional control rates, distant metastasis-free survival rates, overall survival rates and long-term morbidities were not documented in our study.

Our study suggests excellent tolerability of hypofractionated RT for acute dermatitis in our populations. Considering this question has not been explored from our part of the world in both subsets of patients. Our study also evaluated the severity of acute dermatitis and its relation with time throughout the course of radiation therapy and follow-up.

## Conclusion

Hypofractionated radiation therapy is a very promising regimen because of the shorter overall treatment time which reduces the socio-economic burden, not only for patients but also for radiotherapeutic institutions in LMICs. Further building upon this database will help find outcomes related to disease control and late effects concerning the dose of radiation therapy.

## Funding

No funding was received for this article.

## Conflicts of interest

The authors declare no conflicts of interest in this work.

## Authors’ contributions

Dr Yumna developed the concept and design of the study, acquired data for analysis and interpreted the data.

Dr Hammad and Dr Fatima drafted the article.

Dr Rabia, Dr Maria and Dr Sehrish revised the article critically for important intellectual content.

Dr Bilal and Dr Nasir finally approved the version to be published.

## Figures and Tables

**Table 1.** Patient and treatment characteristics

Characteristic	*n* (%)
**Patients**	92
**Age (years)** **Median (range)** **>50 y**ea**r**s **<50 y**ea**r**s	53 40 (43.5) 52 (56.5)
**Laterality** **Right** **Left**	46 (50) 46 (50)
**Tumour type** **IDC** **ILC** **Ductal Carcinoma In Situ (DCIS)**	88 (95.7) 02 (2.2) 02 (2.2)
**Pathologic T stage** **Tis** **T1** **T2** **T3** **T4**	02 (2.2) 02 (2.2) 53 (57.6) 27 (29.3) 08 (8.7)
**Pathologic N stage** **N0** **N1** **N2** **N3**	23 (25.0) 28 (30.4) 21 (22.8) 20 (21.7)
**Stage** **0** **I** **II** **III**	02 (2%) 02 (2%) 37 (42%) 51 ( 64%)
**Surgery** **BCS** **Mastectomy**	31 (33.7) 61 (66.3)
**Treatment technique** **2D** **3D** **IMRT**	02 (2.2) 44 (47.8) 46 (50)
**Boost** **Yes** **No**	83 (90.2) 08 (8.7)
**Chemotherapy** **Yes** ** No**	89 (96.7) 03 (3.3)
